# Cashew Nut Quality as Influenced by Microwave Heating Used for Stored Grain Insect Control

**DOI:** 10.1155/2014/516702

**Published:** 2014-10-09

**Authors:** Ipsita Das, Narendra G. Shah, Girish Kumar

**Affiliations:** ^1^Department of Electrical Engineering, Indian Institute of Technology Bombay, Powai, Mumbai-400076, India; ^2^Centre for Technology Alternatives for Rural Areas, Indian Institute of Technology Bombay, Powai, Mumbai-400076, India

## Abstract

The objective of this work is to investigate the effect of microwave power levels (240, 360, and 480 W) and exposure time (30, 60, 90, 120, 180, and 240 s) on various properties of cashew nuts being used for disinfestation. The nuts were analyzed for moisture content, temperature rise, colour, free fatty acid (FFA) and peroxide value (PV). Experiments were conducted according to the response surface methodology. Increase in microwave power level and exposure time caused a decrease in moisture content, increase in temperature, and change in colour. Microwave treatment to target temperatures of 50–55°C (unfavorable for insect survival) made the PV of cashew nut decrease to 1.10 to 1.66 meq O_2_/Kg (from an initial value of 2.08 ± 0.05) and FFA value to 0.11 to 0.51% (from an initial value of 0.68 ± 0.03). Though PV and FFA values of microwave treated cashew nut were found to increase after 6 months of storage at room conditions, the values were within the limits for acceptable quality. Microwave treated cashew nuts were free from infestation and rancidity even after 6 months of storage while the untreated nuts were found to be heavily infested at the end of 1 month of storage.

## 1. Introduction

Cashew nuts are globally consumed for their desirable nutritional and sensory attributes [1]. Cashew nuts are good source of proteins (~20%), carbohydrates (~23%), and fats (~45%) [2]. India is the second largest producer and exporter of cashew nuts in the world accounting for 40% of world cashew production and contributing about 7% of the total export earnings. During the year 2011-2012, India exported 132,000 MT of cashew kernels valued at Rs 4400 crores (nearly 700 million dollars). USA, UAE, the Netherlands, UK, Japan, France, and Canada are the major international buyers of Indian cashews (http://www.cashewindia.org/php/cepcContents.php?CatID=29). However, cashew nuts are very susceptible to infestation by molds, insects, larvae, and so forth [3]. Insects cause considerable damage to nuts with weight and nutritional losses reducing yield and market value. It has also been reported that any abrasion in the nuts because of insect attack makes the fats liable to become rancid, and thereby integrity of kernels is lost [4]. Almost all countries have imposed a zero tolerance to insects on imported food products. Several methods have been suggested to control insect pests in agricultural commodities. Chemical fumigation is being used extensively for stored food grains. Because of increasing public concern about adverse impacts of chemical fumigation on humans and the environment, there is an increased interest in developing technically effective and environmentally sound quarantine methods especially thermal ones. An important key to develop successful thermal treatments is to balance needs for a complete kill of insects with a minimal thermal impact on product quality. The common difficulties in using conventional hot air disinfestation method are the slow heating rate, nonuniform temperature distribution, and possible heat damage to heat-sensitive commodities [5–7]. Though irradiation process is an efficient method of disinfestation, users are reluctant to accept the process due to safety aspects associated with the irradiation and also not available everywhere [8]. Controlled atmospheres are always being seen as average alternative because of longer treatment time and not being suitable for dealing with high level of infestation [9].

Microwave heating is increasingly being recognized as an alternative method for killing insects in stored grain because of several advantages such as rapid heating, minimal impact on the environment, and existence of no harmful chemicals in treated produce. In India, little emphasis is being given to use of microwave heating technology for disinfestations application. Microwave radiation with good penetrability can kill pests existing inside the grain kernels (if any) thereby taking care of latent infestation. Insect management is considered successful if infestation of insects in food grains or nuts could be prevented as prevention is safer, easier, and less expensive than controlling them [5]. The present research is aimed at studying the disinfestation process (as a method of prevention) of cashew nut using microwave energy. However, improper application of microwave energy can cause irreversible changes in the quality parameters of the product. Microwave heat treatment of whole kernels needs to be properly controlled because it not only contributes to change in flavour, aroma, and colour but also affects the composition. Though there are many research papers on effect of heating on changes in colour and nutritional compositions of peanuts, hazelnuts, macadamias, and so forth [10–17], very little information is available on properties and storability study of cashew nut subjected to heating for disinfestation process. The objective of the paper is to study the effect of microwave power level and exposure time on moisture content, surface temperature rise, colour change, and compositional analysis (peroxide value and free fatty acid) of cashew nut.

## 2. Materials and Methods

Cashew nuts were procured from the market and any foreign matter was manually removed before its use in the experiment. Cashew nuts were graded by size to eliminate the variations with respect to exposed surface area. About 200 g of fresh samples was taken for each experiment. A domestic microwave oven (LG Make, Intellowave, 3850w2G031A) with maximum output of 900 W at 2450 MHz was used for the experiments. The oven had a facility to adjust both microwave power level and exposure time. A container made of polycarbonate with provision to spread the samples uniformly was placed inside the microwave oven cavity on a rotating turn table for an even absorption of microwave energy. The disinfestation experiments followed a factorial design.  Table 1 shows the experimental design parameters. The ranges of experimental parameters were selected based on preliminary trials. The independent variables considered were microwave power level (240, 360, and 480 W) and exposure time (30, 60, 90, 120, 180, and 240 s). The response functions (dependent variables) were temperature rise (Δ*T*), colour change (Δ*E*), water activity (*a*_*w*_), peroxide value (PV), and free fatty acid (FFA). Each treatment (power and exposure time combination) was replicated 3 times.

### 2.1. Quality Attributes

The various quality parameters (Table 1) were evaluated for fresh and treated samples using standard procedures. Standard hot air oven method [18] was used to determine the initial moisture content of cashew nut. Nuts were first ground and then 2-3 g flour samples were placed in petri dishes and kept in a hot air oven at 80°C for 24 hours. There were three replicates for each measurement.

(a) Moisture loss: for moisture loss analysis, fresh cashew nuts were exposed to different power levels and exposure time. The weight loss of sample with time for each run was recorded. The moisture content (%wb) was calculated by dividing the change in the weight by initial weight of the product.
(b) Water activity (*a*_*w*_): the water activity of samples was determined as a measure of storage stability using water activity meter (Decagon Devices, Inc., USA, Model number: CX-3TE). The instrument uses a dielectric humidity sensor to measure the water activity of a sample. Two replicates were made for each sample and 3 readings for each replicate were taken and the mean was used for analysis.
(c) Colour change (Δ*E*): the colour values of the samples measured with a Hunter Lab Color meter (USA, Model number: Color flex 45/0) were expressed as *L* ^*^ (whiteness or brightness/darkness), *a* ^*^ (redness/greenness), and *b* ^*^ (yellowness/blueness). Colour change (Δ*E*) was calculated using (1). The lesser the Δ*E* value is, the more it is closer to untreated/fresh sample:
  (1) ΔE=[(L−L∗)2+(a−a∗)2+(b−b∗)2]0.5.
 Untreated cashew nuts were taken as ideal sample having *L* ^*^, *a* ^*^, and *b* ^*^ values of 70.37, 1.61, and 16.47, respectively.
(d) Surface temperature rise (Δ*T*): surface temperatures of nuts were measured using noncontact infrared thermometer (DIT 130, range −32 to 380°C, Germany). A microwavable rectangular box was used to hold 200 g of sample. The grain samples were immediately kept in the box after the treatment and surface temperatures were measured. Ambient room temperature (29°C) was used as the initial sample temperature for each test.
(e) Compositional analysis: the compositional analysis of treated samples was carried out to determine PV and FFA according to AOAC standards [19] [965.33, 940.28].
(f) Storability study: at the end of the microwave treatment, samples were left on the sample tray for 2 to 3 min to allow the heat to redistribute in nuts and impart added lethality, before being transferred into air tight container for storability study. The treated nuts were analyzed after 6 months of storage for appearance of infestation and change in composition, if any. Also the storability of fresh cashew kernels was studied under ambient conditions.

### 2.2. Analysis of Data

The respective ranges of each experimental parameters (Table 1) were selected based on preliminary trials. The independent variables considered were microwave power with three levels and exposure time with six levels. Response surface methodology (RSM) was used to understand the interactions of microwave power level and exposure time on the quality of treated nuts. The qualities of treated nuts studied were colour change, temperature rise, and compositional analysis (PV and FFA). The second-order polynomial model (2) was used for analysis of experimental data and to relate response function to independent variables. The coefficients of the polynomial were represented by *b*_0_ (constant term), *b*_1_ and *b*_2_ (linear effects), *b*_11_ and *b*_22_ (quadratic effects), and *b*_12_ (interaction effects). The analysis of variance (ANOVA) tables were generated and the effect of regression coefficients of individual linear, quadratic, and interaction terms was determined using design expert software (version 9, STAT-EASE, Inc., USA.15.1.1.0). The significance of all the terms in the polynomial was judged statistically by computing the *p* value at 1% and 5% levels of significance:

(2) Y=b0+b1P+b2T+b12PT+b11P2+b22T2.

## 3. Results and Discussions

### 3.1. Moisture Loss and Water Activity

**Figure 1:**
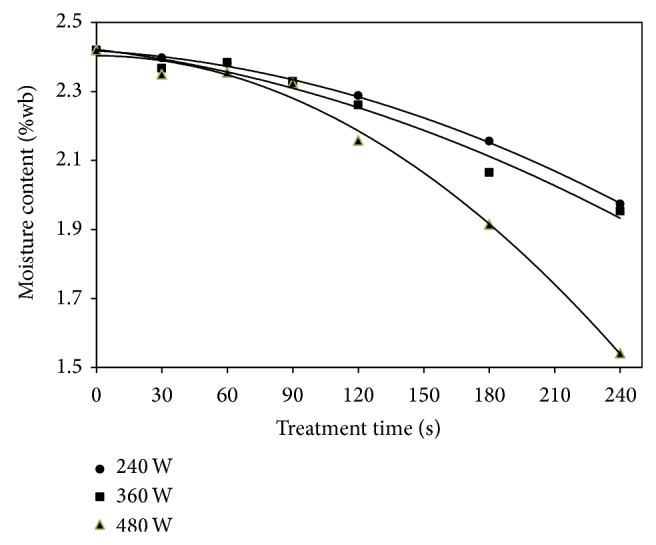
Variation of moisture content with exposure time for different power levels of cashew nuts.

Figure 1 shows the change in moisture content of cashew nut at different microwave power levels and exposure time. With increase in exposure time, there was a decrease in moisture content regardless of level of power. The initial moisture content (IMC) of fresh cashew kernel was found to be 2.5% ± 0.2. With increase in exposure time (from 30 to 240 s), there was a decrease in moisture content of cashew nut from IMC of 2.5% to 1.97, 1.95, and 1.54% (wet basis) at power levels of 240, 360, and 480 W, respectively (Table 2). The water activity was found to be in the range of 0.37 to 0.49 and no significant difference was noted among treatments. Foods having a water activity between 0.3 and 0.5 are normally considered to be shelf-stable dried foods [20]. The water activity values of fresh and treated cashew nut for different operating condition are listed in Table 2.

### 3.2. Colour Change (Δ*E*)

**Figure 2:**
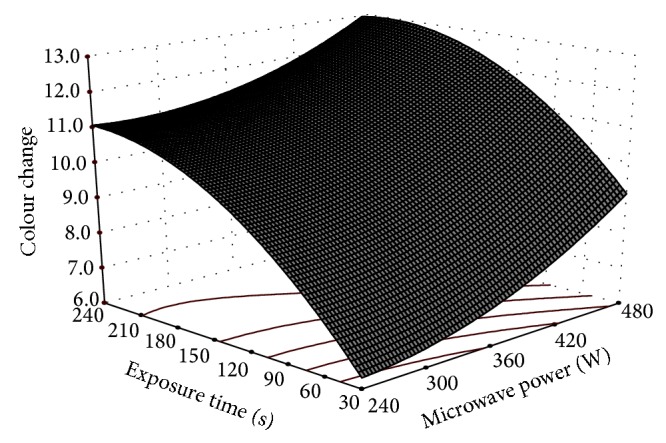
Effect of microwave power and exposure time on color change of cashew nuts.

The value of color change (Δ*E*) of treated cashew nuts varied between 6.11 and 12.96 (Table 2) for different operating conditions. ANOVA (Table 3) indicates linear terms of microwave power level and exposure time (at *p* ≤ 0.01) and quadratic terms of process variable (at *p* ≤ 0.05) are significantly affecting the color change. While the effect of interaction term was found to be nonsignificant. By neglecting the nonsignificant terms the following equation describes the effect of power level and exposure time on color change of cashew nut:

(3) ΔE=10.48+1.17P+2.13T+0.61P2−1.19T2,

where (Δ*E*) is the colour change; *P* is the microwave power level (W); *T* is the exposure time (s). The positive coefficients of the first-order terms of microwave power level and exposure time (3) indicate that colour change increases with increase of these variables. Coefficient of determination *R* ^2^ for this model was found to be 0.97 and a value of 0.70 and greater indicates a good model fit [21]. Microwave power level has maximum effect on colour change as indicated by *F* value and regression coefficient. Other researchers have also observed that food products such as garlic cloves, date, and okra get darker when higher air temperature and microwave power level are used during heating [22–24]. This colour inducing effect is primarily due to nonenzymatic browning of Maillard type reaction [25]. The variation of colour change with microwave power level and exposure time has graphically been presented in the 3D plot (Figure 2).

### 3.3. Temperature Rise (Δ*T*)

The final kernel temperature is a key factor for both insect mortality and cashew kernel quality. The measured values of temperature rise for different combinations of process parameters are presented in Table 2 and found to vary between 35.6 and 93.1°C. It has been reported [26] that the temperature between 50 and 55°C is lethal for insects. The heating time was found to be 90 to 120 s for kernels depending upon the power level and exposure time. The heating time is defined as the time taken by nuts to reach the target temperature, that is, 50–55°C. The second-order polynomial equation (4) was fitted to the experimental data and tested for adequacy through ANOVA:

(4) ΔT=62.32+5.15P+21.82T+2.82PT.

**Figure 3:**
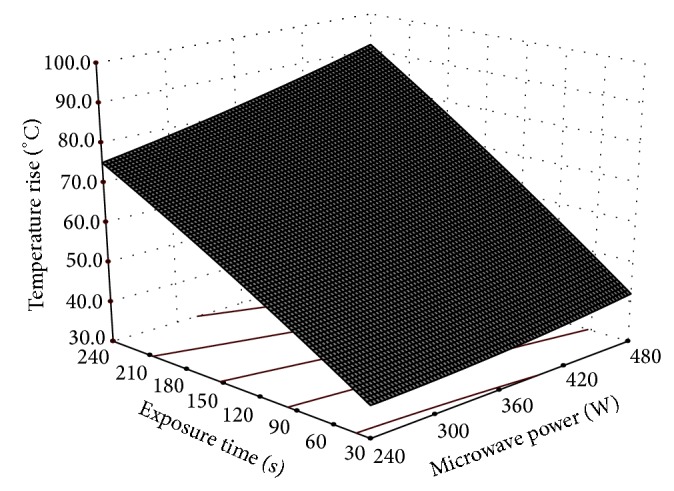
Effect of microwave power and exposure time on temperature rise of cashew nuts.

Temperature of nuts was found to increase linearly with increase in power level and exposure time. Exposure time has maximum effect on temperature rise as compared to power level as indicated by *F* value and regression coefficient. The experimental data fitted the second-order polynomial equation well as indicated by high *R* ^2^ value, that is, 0.96, and low COV value at 5.1% (considered best fit if the value is less than 10% [24]). ANOVA (Table 3) indicated that temperature rise was significant at 1% level on linear terms of power level and exposure time. Quadratic terms were found not to have any effect while interaction term was having significant effect at 5% level.  Figure 3 shows the effect of process parameters on temperature rise.

### 3.4. Compositional Analysis of Cashew Nut

**Figure 4:**
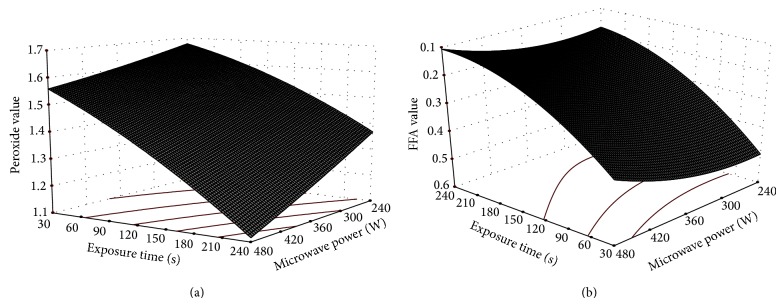
Effect of microwave power and exposure time on (a) PV and (b) FFA values of cashew nuts.

Cashew nut contains substantial quantities of polyunsaturated fatty acids (~55%) and thus is susceptible to oxidative and hydrolytic rancidity, which in turn produces undesirable volatile compounds and off-flavors which limit the shelf life [27]. Chemical analyses for assessing oxidative rancidity and hydrolytic rancidity include PV and FFA, respectively. According to UN specifications, the maximum tolerated values of PV and FFA of cashew kernel have to be less than 5.0 meq O_2_/Kg of oil and 1.0%, respectively [28]. The values of PV and FFA for untreated (fresh) cashew nut were 2.08 ± 0.05 meq O_2_/Kg of oil and 0.68 ± 0.03% at the start of the experiment. Immediately after the microwave exposure, the values of PV and FFA dropped to the ranges of 1.10 to 1.66 and 0.11 to 0.51, respectively, for the range of independent variables studied. Both PV and FFA decrease with increase in power level and exposure time. The effect of microwave power level and exposure time on PV and FFA is presented in Figures 4(a) and 4(b). ANOVA (Table 3) indicates that both microwave power level and exposure time (linear terms) have significant effect on PV and FFA values (*p* ≤ 0.05):

(5) $$\begin{array}{c} \text{PV}=0.46-0.093P-0.29T-0.036PT,\\ \text{FFA}=0.26-0.046P-0.15T-0.038P^{2}+0.064T^{2}. \end{array}$$

The PV and FFA values of microwave treated cashew nut were found to increase slightly (Table 4) after 6 months of storage at room conditions, yet the values were within the acceptable limits. An increase in peroxide value always indicates the onset of oxidative rancidity [29]. Treated cashew nuts could be stored for 6 months at room temperature without any infestation and adverse effect on quality. The infestation was noticed only in the sample, which had been treated with low microwave power level (i.e., 240 watt) and low exposure time (30 s) after 4 months of storage. In contrast, fresh cashew kernels were heavily infested at the end of 1 month of storage. So, the microwave treatment might have inactivated the lipase enzyme responsible for the formation of FFA, thereby resulting in prevention of deterioration during storage [30]. It has also been reported by Hamilton [31] that moisture content and lipase activity in the nuts can be controlled by thermal process such as heating and roasting.

## 4. Conclusion

Our technology, that is, short time microwave treatment, is a potential means of replacing other existing quarantine methods because of several advantages such as shortening of treatment time, better quality retention, and energy minimization. The technology involves treating the cashew nut with microwave energy for a short time to prevent infestation as well as rancidity during storage. Microwave radiation with good penetrability can kill pests existing inside the nut kernels (if any) thereby taking care of latent infestation. The effect of microwave power levels (240–480 W) and exposure time (30–240 s) on moisture content, water activity, colour change, temperature rise, and compositional analysis (PV and FFA values) of cashew nuts was investigated. Increases in microwave power level and exposure time caused a decrease in moisture content and increase in temperature and colour change. Untreated cashew nuts exhibited higher peroxide and FFA values than short time microwave treated kernels under equivalent conditions. We could increase the storability of nuts with no adverse effects on product quality for 6 months under ambient conditions as compared to untreated control samples which got infested/spoiled at the end of 1 month of storage. Short time microwave treatment of cashew nut did not promote rancidity which in turn helped in increased shelf life.

## Figures and Tables

**Table 1:** Experimental details (process parameters and their levels).

Factors	Levels	Variables
Microwave power (W) Exposure time (s)	240, 360, and 480 30, 60, 90, 120, 180, and 240	(i) Moisture loss (ii) Water activity (iii) Temperature rise (iv) Colour change (v) Peroxide value (vi) Free fatty acid value

**Table 2:** Values of different quality parameters of microwave (MW) pretreated cashew nut.

S. number	Power level (W)	Exposure time (s)	Moisture content (%wb)	Water activity *a*_*w*_	Colour change (Δ*E*)	Temperature rise, °C (Δ*T*)
1	240	30	2.40	0.489	6.11	35.63
2		60	2.38	0.469	7.81	46.20
3		90	2.32	0.498	8.37	50.27
4		120	2.29	0.430	10.11	57.40
5		180	2.16	0.433	10.54	63.80
6		240	1.97	0.421	11.00	75.30

7	360	30	2.37	0.412	7.12	37.07
8		60	2.38	0.418	8.18	48.70
9		90	2.33	0.418	8.91	52.40
10		120	2.26	0.357	10.82	59.43
11		180	2.07	0.350	11.02	67.33
12		240	1.95	0.347	11.53	87.10

13	480	30	2.35	0.402	9.53	40.67
14		60	2.36	0.440	10.51	52.43
15		90	2.32	0.444	10.80	58.43
16		120	2.16	0.362	11.81	67.47
17		180	1.91	0.401	12.88	73.50
18		240	1.54	0.371	12.96	93.10

**Table 3:** ANOVA for different quality attributes of microwave treated cashew nut.

Source of variation	*F* value	*p* value
(a) Colour change		
Model∗∗	90.02	<0.0001
Power (*P*)∗∗	117.11	<0.0001
Exposure time (*T*)∗∗	28.53	<0.0001
*P* × *T* ^ns^	3.38	0.091
*P* ^2^^*^	10.91	0.006
*T* ^2^^*^	29.3	0.0002

(b) Temperature rise		
Model∗∗	92.31	<0.0001
Power (*P*)∗∗	32.92	<0.0001
Exposure time (*T*)∗∗	427.27	<0.0001
*P* × *T* ^*^	4.77	0.049
*P* ^2^ ^ns^	0.31	0.589
*T* ^2^ ^ns^	0.36	0.558

(c) Peroxide value		
Model∗∗	66.16	<0.0001
Power (*P*)∗∗	82.25	<0.0001
Exposure time (*T*)∗∗	244.27	<0.0001
*P* × *T* ^*^	5.85	0.0324
*P* ^2^ ^ns^	0.11	0.7418
*T* ^2^ ^ns^	2.26	0.1582

(d) Free fatty acid		
Model∗∗	41.26	<0.0001
Power (*P*)∗	22.02	0.0005
Exposure time (*T*)∗∗	166.83	<0.0001
*P* × *T* ^ns^	0.99	0.338
*P* ^2^^*^	5.20	0.042
*T* ^2^^*^	10.53	0.007

^**^Significant at 1% level (*p* value less than 0.0001).

∗Significant at 5% level (*p* value less than 0.050).

^ns^Nonsignificant.

**Table 4:** Compositional analysis of cashew nut immediately after microwave treatment and after 6 months of storage under room conditions.

S. number	Power level (W)	Exposure time (s)	Peroxide value, meq O_2_/Kg		FFA, %	
			Immediately after treatment	After 6 months of storage	Immediately after treatment	After 6 months of storage
	Fresh/untreated sample		2.08 ± 0.05		0.68 ± 0.03	
1	240	30	1.66	1.86	0.51	0.58
2		60	1.65	1.84	0.39	0.44
3		90	1.66	1.73	0.34	0.39
4		120	1.54	1.70	0.30	0.31
5		180	1.44	1.62	0.22	0.24
6		240	1.42	1.45	0.16	0.18

7	360	30	1.63	1.85	0.46	0.51
8		60	1.60	1.81	0.38	0.42
9		90	1.54	1.72	0.33	0.31
10		120	1.45	1.57	0.30	0.27
11		180	1.38	1.42	0.24	0.21
12		240	1.21	1.22	0.15	0.17

13	480	30	1.53	1.74	0.44	0.46
14		60	1.51	1.72	0.24	0.27
15		90	1.48	1.63	0.22	0.24
16		120	1.39	1.42	0.20	0.19
17		180	1.31	1.31	0.14	0.15
18		240	1.10	1.12	0.11	0.11
